# The effect of CPAP on the upper airway and ventilatory flow in patients with obstructive sleep apnea

**DOI:** 10.1186/s12931-023-02452-z

**Published:** 2023-05-31

**Authors:** Eli Van de Perck, Elahe Kazemeini, Karlien Van den Bossche, Marc Willemen, Johan Verbraecken, Olivier M Vanderveken, Sara Op de Beeck

**Affiliations:** 1 Faculty of Medicine and Health Sciences, Department of Translational Neurosciences, University of Antwerp, Universiteitsplein 1 – D.T.494, Wilrijk, Belgium; 2 Department of Otorhinolaryngology, Head and Neck Surgery, Antwerp University Hospital, Edegem, Belgium; 3 Multidisciplinary Sleep Disorders Centre, Antwerp University Hospital, Edegem, Belgium; 4 Department of Pulmonology, Antwerp University Hospital, Edegem, Belgium

**Keywords:** Airflow, DISE, Endoscopy, Epiglottis, Larynx, OSA, Positive airway pressure.

## Abstract

**Background:**

Continuous positive airway pressure (CPAP) is the mainstay of treatment for obstructive sleep apnea (OSA). However, data about its effect on the upper airway, especially the epiglottis, are scarce. The aim of this study was to investigate the changes in upper airway dimensions and inspiratory flow in response to incremental pressure levels.

**Methods:**

This is a secondary analysis of a prospective clinical trial in which patients with moderate to severe OSA underwent drug-induced sleep endoscopy with simultaneous recordings of flow and mask pressure. CPAP was titrated in small increments. For each pressure level a representative 3-breath segment was selected to determine specific flow features. The corresponding endoscopic footage was reviewed to assess the degree of upper airway collapse in a semi-quantitative manner.

**Results:**

A total of 214 breath segments were obtained from 13 participants (median [Q1–Q3]; apnea-hypopnea index, 24.9 [20.1–43.9] events/h; body mass index 28.1 [25.1–31.7] kg/m²). CPAP significantly increased cross-sectional dimensions of the soft palate, lateral walls and tongue base, but not of the epiglottis, and induced epiglottis collapse in one subject. Increased pressure improved peak inspiratory flow and median ventilation in all patients, even in the presence of persistent epiglottis collapse.

**Conclusion:**

CPAP does not effectively address epiglottis collapse in patients with OSA. However, it normalizes inspiratory flow regardless of its effect on the epiglottis. This clinical trial was registered on January 18th, 2020 on ClinicalTrials.gov with identifier NCT04232410.

## Introduction

Continuous positive airway pressure (CPAP) therapy counteracts upper airway collapse by acting as a pneumatic splint and is regarded as the mainstay of treatment for adults with moderate to severe obstructive sleep apnea (OSA) [1, 2]. Individual characteristics are often not taken into account when initiating CPAP because of its optimal efficacy across patient populations. However, previous research has indicated that CPAP is ineffective in the presence of collapse of the epiglottis [3–6]. These studies have one or more limitations that may have affected their accuracy and applicability: (1) they were purely observational and failed to demonstrate a causal relationship between epiglottis collapse and CPAP failure; (2) they included patients with neurological comorbidities; and (3) they did not directly measure the amount of CPAP applied to patients.

This study sought to determine the effect of incremental CPAP levels on upper airway structures, particularly the epiglottis, during drug-induced sleep endoscopy (DISE). In addition, we evaluated whether CPAP-resistant collapse impacts ventilatory flow.

## Methods

This study is a secondary analysis of a prospective trial (NCT04232410) that was approved by the ethics committee of the University of Antwerp and Antwerp University Hospital. Written informed consent was obtained from all participants. The parent trial included patients with moderate to severe OSA who required evaluation by DISE.

The study design and set-up were described in detail by Kazemeini et al. [7]. All patients underwent conventional DISE while wearing a modified nasal mask connected to a calibrated pneumotachograph (ventilatory flow, L/min) and differential pressure transducer referenced to the surrounding atmosphere (mask pressure, P_mask_, cmH_2_O). Sleep depth was monitored by electroencephalography, electrooculography, and chin electromyography. Respiratory effort was measured using an esophageal pressure catheter and inductive plethysmography. All signals, synchronized with endoscopic footage, were captured using an Alice LDx6 polysomnography system (Philips Respironics, Murrysville, PA, USA).

Artificial sleep was induced by intravenous bolus injection of midazolam and maintained by target-controlled infusion of propofol. Additionally, glycopyrrolate was administered to reduce salivation. Once stable sleep was achieved, positive pressure was applied by a modified CPAP device (Pcrit3000, Philips, Amsterdam, Netherlands) and adjusted in increments of 1 to 2 cmH_2_O until resolution of flow limitation in each individual. Endoscopic observations were made in the naso- and oropharynx with the patients lying supine.

For each CPAP level, we selected one breath segment consisting of three consecutive, high-quality breaths, accompanied by clear endoscopic footage. Subsequently, the degree of collapse was assessed for each corresponding segment at the levels of the soft palate, lateral walls, tongue base and epiglottis using a semi-quantitative scale (i.e. 0%, 25%, 50%, 75%, 100%). All measurements were made by one experienced examiner (EVDP) who was blinded to baseline characteristics and CPAP levels.

Two features were extracted from the pneumotachograph signal using custom-made software (MATLAB, The MathWorks, Natick, MA, USA) to define flow limitation: peak inspiratory flow (PIF), which was calculated as the difference between maximum and average inspiratory flow, and median inspiratory ventilation (V_i_) [7]. P_mask_ was averaged throughout the entire respiratory cycle.

The primary analysis evaluated the relationship between P_mask_ and upper airway dimensions. In a secondary analysis, trends in PIF and V_i_ were assessed. Linear mixed regression models were used to account for within-subject correlation. All data are presented as median (Q1─Q3) unless otherwise specified. Statistical significance was considered if P < .05.

## Results

All 13 participants of the parent study were eligible for this secondary analysis. Table 1 summarizes their clinical characteristics and baseline DISE data. Of the 345 breath segments obtained, 131 were excluded due to flaws in the flow signal, leaving 214 segments for further analysis (median 16 [11─23] per subject). Sixty-seven of them were captured in the nasopharynx and 147 in the oropharynx.

**Table 1:** Patient characteristics

Characteristic	Value
*Demographics and anthropometrics*	
Men, No. (%)	13 (100)
Age, year	51 (49–56)
BMI, kg/m²	28.1 (25.1–31.7)
Neck circumference, cm	42 (40─43)
*Polysomnographic parameters*	
AHI, events/h	24.9 (20.1─43.9)
Obstructive AHI, events/h	24.5 (19.9─43.3)
Supine AHI, events/h	42.9 (32.2─60.2)
Apnea index, events/h	1.2 (0.4─7.0)
ODI, events/h	18.5 (9.9─28.3)
*Baseline DISE collapse*	
Soft palate, No. (%)	12 (92)
Partial	5 (38)
Complete	7 (54)
Lateral walls, No. (%)	10 (77)
Partial	5 (38)
Complete	5 (38)
Tongue base, No. (%)	7 (54)
Partial	5 (38)
Complete	2 (15)
Epiglottis, No. (%)	2 (15)
Partial	1 (8)
Complete	1 (8)

Continuous variables are presented as median (Q1─Q3). Abbreviations: AHI = apnea-hypopnea index; BMI = body mass index; DISE = drug-induced sleep endoscopy; ODI = oxygen desaturation index.

### Primary analysis

**Fig. 1:**
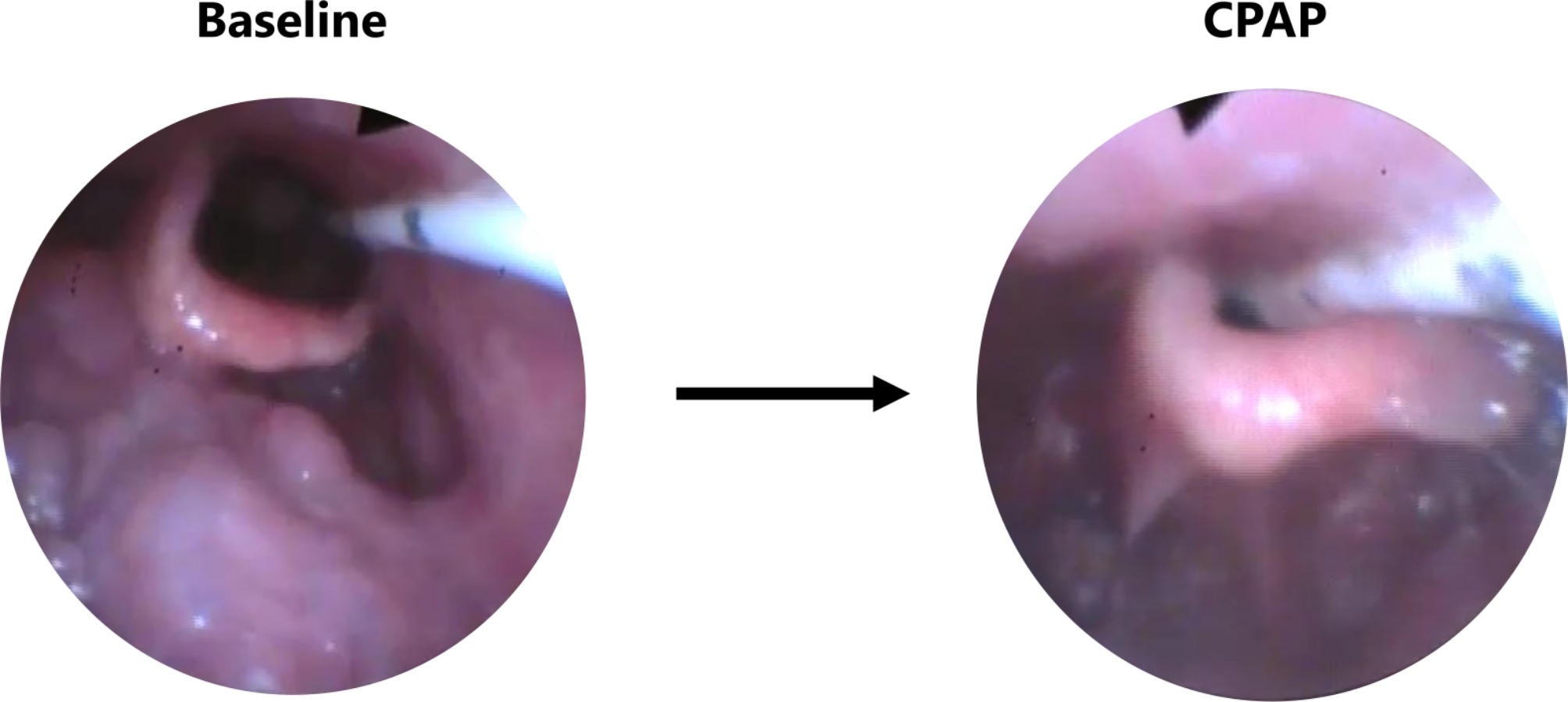
Example of CPAP-induced epiglottis collapse

**Fig. 2:**
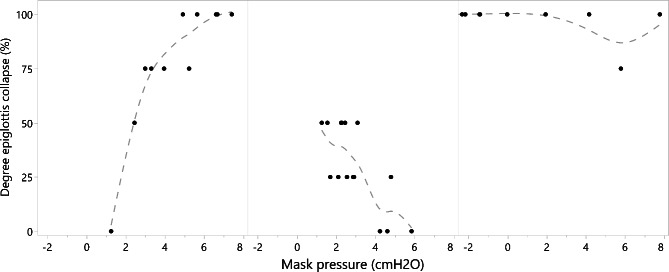
Effect of CPAP in subjects with epiglottis collapse The first subject developed epiglottis collapse starting from a P_mask_ of 2.4 cmH_2_O. In the second subject, a P_mask_ of around 2.5 cmH_2_O effectively eliminated partial epiglottis collapse. The last subject exhibited persistent epiglottis collapse despite high pressure levels

Fifty-four breath segments were associated with collapse (defined as ≥ 50% narrowing) of the soft palate, 47 with collapse of the lateral walls, 47 with collapse of the tongue base, and 26 with collapse of the epiglottis. Increasing P_mask_ resulted in significant cross-sectional improvements at the levels of the soft palate, lateral walls and tongue base (Table 2). However, there was no relationship between P_mask_ and epiglottic dimensions. CPAP had varying effects on the epiglottis: it resolved partial collapse in one subject, it did not affect complete collapse in another subject, and it even promoted collapse in a third subject (Figs. 1 and 2). CPAP effectively eliminated collapse at the other upper airway levels, although three patients had persistent collapse of the soft palate. An average P_mask_ of 4.7, 4.3 and 4.4 cmH_2_O was needed to overcome collapse at the level of the soft palate, lateral walls and tongue base, respectively.

**Table 2:** Linear mixed regression analysis correlating mask pressure and upper airway dimensions

Site	β coefficient	95% CI	P-value
Soft palate	-9.5	-11.4 to -7.5	< 0.001
Lateral walls	-5.3	-6.4 to -4.2	< 0.001
Tongue base	-5.3	-6.3 to -4.3	< 0.001
Epiglottis	0.2	-0.5 to 0.9	0.54

Separate linear models were fitted for each upper airway level with random patient effect to account for non-independence of the observations.

### Secondary analysis

**Fig. 3:**
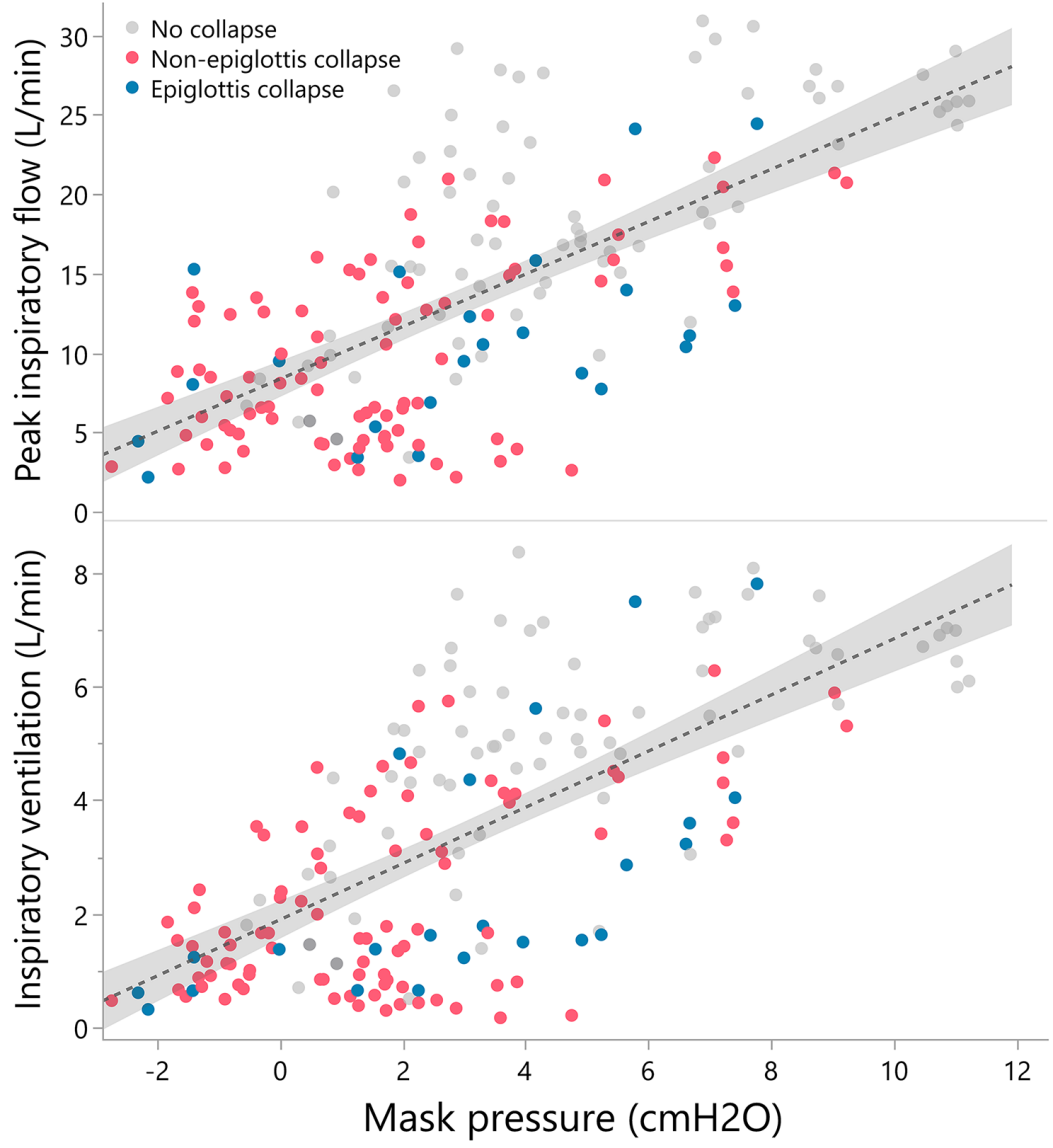
Relationship between mask pressure, inspiratory flow and upper airway collapse Collapse was defined as ≥ 50% narrowing of the upper airway. Fitted lines with 95% confidence intervals represent all breath segments

P_mask_ was positively associated with both PIF (β, 2.5; 95% CI 2.3─2.7; *P* < .001) and V_i_ (β, 0.75; 95% CI, 0.68–0.80; *P* < .001). Ventilation under CPAP was affected by collapse at the levels of the soft palate, lateral walls and tongue base (all *P* < .001 for PIF and V_i_ when corrected for P_mask_), but not by collapse of the epiglottis (*P* = .38 and *P* = .34, respectively, Fig. 3). There was no significant difference in PIF (*P* = .77) and V_i_ (*P* = .92) between patients with persistent epiglottis collapse and their counterparts based on Wilcoxon’s test.

## Discussion

This is the first study to assess the effects of CPAP on upper airway dimensions and inspiratory flow using gold standard measurements [8]. The main conclusion is that epiglottis collapse is not alleviated by CPAP, as opposed to other upper airway structures. However, this does not seem to hamper the effectiveness of CPAP in improving inspiratory flow.

Our findings are in line with previous research indicating that epiglottis collapse is refractory to CPAP and often requires pressure levels above 10 cmH_2_O [4, 5]. Additionally, our study included one individual in whom CPAP adversely induced collapse of the epiglottis, which supports previous observations from case series [3, 9, 10]. This effect of CPAP can be attributed to the anatomy of the epiglottis, which is orientated in the opposite direction of inspiratory flow and is tightly connected by ligaments to the hyoid and thyroid [11]. CPAP has little impact on these structures, and therefore, epiglottic movement may be limited. However, when this suspensory apparatus degenerates [10, 12], the epiglottis may become lax and susceptible to collapse when exposed to positive pressure.

In general, CPAP has the greatest impact on the pharyngeal lateral walls which are more compliant than the soft palate and tongue base [5, 13]. Nevertheless, we found similar pressure requirements for all three upper airway levels. Persistent collapse was only seen at the soft palate and epiglottis. This is in accordance with research of Dieleman et al. who applied CPAP during DISE to identify causes of CPAP failure [6]. Together, these findings suggest that both the soft palate and epiglottis are important areas to consider when dealing with suboptimal CPAP results.

Interestingly and unexpectedly, CPAP normalized PIF and V_i_ in all patients regardless of its effect on the epiglottis. This finding challenges the notion that epiglottis collapse is a contra-indication for CPAP therapy. However, it is important to bear in mind that epiglottis collapse under CPAP may cause discomfort, particularly a choking sensation, thereby reducing adherence. This has been recently demonstrated by a case-control study in which patients with epiglottis collapse were less adherent and more likely to terminate CPAP than controls [14]. Therefore, other treatment strategies that are known to be effective, such as oral appliances and positional interventions, should be considered as well in patients with primary epiglottis collapse [15, 16].

A particular strength of this study were the direct measurements of P_mask_ which reflects effective CPAP. Air leakage during CPAP is not uncommon, especially in research settings with modified masks and simultaneous endoscopy, and can result in erroneous observations. Therefore, these measurements allow for more accurate inferences on CPAP-related changes in upper airway dimensions.

This study has some limitations. First and foremost, due to the invasive nature of the measurements, the sample size was rather small, and while female sex was not an exclusion parameter, only males participated in the study. Patients also had less severe OSA compared to previous studies which may explain the lower CPAP requirements in the present study. Secondly, CPAP was titrated until resolution of flow limitation, not upper airway collapse. Therefore, it is possible that even higher pressures would have improved collapse of the epiglottis. Lastly, the study did not include data on clinical efficacy and adherence. It would be interesting to evaluate the relationship between persistent epiglottis collapse and CPAP-intolerance in future studies.

Notwithstanding these limitations, the current study provides insights into upper airway mechanics and collapse patterns that may or may not respond favorably to CPAP. Our findings demonstrate that while CPAP does not effectively address epiglottis collapse, it does resolve flow limitation regardless of its effect on the epiglottis.

## Data Availability

The datasets generated during the current study are available from the corresponding author on reasonable request.

## Abbreviations

CPAP: Continuous positive airway pressure
DISE: Drug-induced sleep endoscopy
OSA: Obstructive sleep apnea
PIF: Peak inspiratory flow
P_mask_: Mask pressure
V_i_: Median inspiratory flow
